# Cumulative Live Birth Rates in Low Prognosis Patients According to the POSEIDON Criteria: An Analysis of 26,697 Cycles of *in vitro* Fertilization/Intracytoplasmic Sperm Injection

**DOI:** 10.3389/fendo.2019.00642

**Published:** 2019-09-19

**Authors:** Yuan Li, Xiaofeng Li, Xiaoyi Yang, Sufen Cai, Guangxiu Lu, Ge Lin, Peter Humaidan, Fei Gong

**Affiliations:** ^1^Institute of Reproduction and Stem Cell Engineering, Basic Medicine College, Central South University, Changsha, China; ^2^Reproductive and Genetic Hospital of Citic-Xiangya, Changsha, China; ^3^Key Laboratory of Stem Cells and Reproductive Engineering, National Health and Family Planning Commission, Changsha, China; ^4^Fertility Clinic, Skive Regional Hospital, Skive, Denmark; ^5^Faculty of Health, Aarhus University, Skive, Denmark

**Keywords:** assisted reproductive technology, poor ovarian response, POSEIDON stratification, low prognosis, cumulative live-birth rate

## Abstract

**Objective:** The POSEIDON criteria are used to stratify patients with low prognosis after assisted reproductive technology (ART) treatment. Since its introduction, there has been no large study about the prognosis of the POSEIDON population. We used the POSEIDON criteria in Chinese women who underwent repeated ART treatment and analyzed the association between POSEIDON criteria and the cumulative live-birth rate (CLBR).

**Methods:** This was a retrospective cohort study of 62,749 women (97,388 cycles) who underwent ART treatment at the Reproductive and Genetic Hospital of CITIC-XIANGYA between January 2014 and June 2017. Among them, 19,781 (31.52%) women fulfilled the POSEIDON criteria, including 26,697 cycles. The optimal and conservative CLBRs within a complete IVF/ICSI treatment cycle were calculated, as well as the CLBRs following repeated ovarian stimulation cycles.

**Results:** In POSEIDON groups 1, 2, 3, and 4, the optimal and conservative CLBRs of three complete consecutive *in vitro* fertilization (IVF)/intracytoplasmic sperm injection (ICSI) cycles were 83.87 and 66.06%, 53.67 and 37.72%, 44.24 and 27.98%, and 14.20 and 9.68%, respectively. The POSEIDON stratification [group 2: odds ratio (OR) = 2.319, 95% confidence interval (CI): 2.131–2.525, *P* < 0.001; group 3: OR = 1.356, 95% CI: 1.005–1.828, *P* = 0.046; group 4: OR = 3.525, 95% CI: 2.774–4.479, *P* < 0.001; all vs. group 1] and ovarian stimulation protocol [gonadotropin-releasing hormone (GnRH) antagonist protocol: OR = 1.856, 95% CI: 1.640–2.100, *P* < 0.001; other protocols: OR = 1.651, 95% CI: 1.155–2.361, *P* = 0.006; both vs. long GnRH agonist protocol] were associated with live birth in the first stimulation cycle. For the second stimulation cycle, the POSEIDON stratification (except POSEIDON group 3) and ovarian stimulation protocol were associated with live birth. A change in ovarian stimulation protocol was not associated with an improvement in the live birth rate.

**Conclusions:** More than 30% of women who undergo IVF/ICSI treatment may be classified as low prognosis. Different reproductive outcomes were observed among the four POSEIDON groups. The most optimal outcomes after three successive cycles of IVF/ICSI treatment were observed in groups 1, 2, and 3.

## Introduction

Management of patients with diminished ovarian reserve (DOR) or poor ovarian response (POR) is a challenge in reproductive medicine. Most women with DOR will ultimately require *in vitro* fertilization (IVF) to be pregnant (1). POR limits the success of assisted reproductive technology (ART) (2). In POR, the number of oocytes, but not their quality, limits the success of ART in these patients (3). A comprehensive evaluation of the ovarian reserve and ovarian response is essential for individualized therapeutic strategy in order to optimize the success rate of ART. Many and inconsistent definitions of POR have been used, preventing the direct comparisons among studies, as well as the generalizability and applicability of the results (4). Unfortunately, despite many efforts, there are currently no tests that can reliably predict the ovarian response in all women undergoing ART treatment (2, 5).

After the Bologna criteria for POR were proposed in 2011 (6), the universal definition of POR helped investigators enroll more homogeneous populations when conducting studies in patients undergoing IVF/intracytoplasmic sperm injection (ICSI). Although the Bologna criteria were thought initially to characterize a homogeneous population, subsequent research showed that this was not the fact, and that the Bologna criteria describe a heterogeneous population with different reproductive outcomes, mainly because the effect of age on oocyte quality was not taken into consideration (7, 8). Thus, the Bologna criteria can be considered as a useful mathematical model, but the criteria did not provide enough clinical recommendations for managing this type of patient (9–11). Recently, in an effort to further refine the Bologna criteria, the Patient-Oriented Strategies Encompassing IndividualizeD Oocyte Number (POSEIDON) was proposed. These criteria stratify patients according to age (and therefore the expected euploidy rate), ovarian biomarkers, and ovarian response if a previous stimulation has been performed (7, 8). Moreover, the concept of POR was changed to low prognosis in order to better reflect the clinical difference between POR and DOR (12, 13). The aim of the POSEIDON stratification was not only to help clinicians counsel and set patient expectation, but also to establish a working plan to reduce the time to pregnancy (7, 8, 12, 13).

The primary goal for a woman undergoing ART is a live birth. The majority of patients will undergo repeated stimulation cycles, especially if they belong to the POSEIDON group of low prognosis. Therefore, the cumulative live-birth rate (CLBR) is a very meaningful parameter of efficacy and reproductive success. Until now no study explored the POSEIDON stratification in a large study population. At our institute, >28,000 IVF/ICSI cycles are performed each year.

In the present study, we retrospectively applied the POSEIDON stratification to patients who underwent repeated IVF/ICSI treatment to better determine the CLBRs according to each POSEIDON subgroups in a real world setting. The results could provide evidence to physicians regarding the potential reproductive prognosis of these patients and also indicate whether changes in ART treatment strategies could result in better outcomes.

## Methods

### Study Design and Patients

This was a retrospective study of women who underwent ART at the Reproductive and Genetic Hospital of CITIC-XIANGYA between January 2014 and June 2017. Women ≥18 years old who underwent IVF/ICSI were included. The exclusion criteria were: (1) women who underwent their first ovarian stimulation treatment before 2014; (2) women with adequate ovarian reserve [antral follicle count (AFC) ≥ 5 and anti-Müllerian hormone (AMH) ≥ 1.2 ng/ml] and optimal ovarian response (>9 oocytes retrieved in the first stimulation cycle); (3) women who had adequate ovarian reserve, but did not receive a standard ovarian stimulation protocol [long gonadotropin-releasing hormone (GnRH) agonist protocol or GnRH antagonist protocol] during their first stimulation cycle; or (4) women who received preimplantation genetic screening or preimplantation genetic diagnosis. This study was approved by the Ethics Committee of the Reproductive and Genetic Hospital of CITIC-XIANGYA (LL-SC-2018-033). The need for individual consent was waived by the committee due to the retrospective character of the study.

### POSEIDON Stratification

The POSEIDON stratification was applied retrospectively to the patients according to the age when the patients received their first ART treatment, and based on AFC, AMH, and the number of oocytes retrieved during the first stimulation cycle (7, 8). The POSEIDON groups 1 and 2 included patients with AFC ≥ 5, AMH ≥ 1.2 ng/ml, and ≤9 oocytes retrieved after standard ovarian stimulation. Younger patients (age < 35 years) were included in POSEIDON group 1, while older patients (age ≥ 35 years) were included in POSEIDON groups 2. The POSEIDON groups 3 and 4 consisted of patients with AFC < 5 or AMH < 1.2 ng/ml. Younger patients (age < 35 years) were included in POSEIDON group 3, while older patients (age ≥ 35 years) were included in POSEIDON groups 4.

### Controlled Ovarian Stimulation Protocol

Standard ovarian stimulation protocol referred to long GnRH agonist protocol or GnRH antagonist protocol. For the long GnRH agonist protocol, on day 20 of the patient's menstrual cycle, 1.5–1.875 mg of GnRH agonist were injected intramuscularly. After 13–20 days, after confirmation of pituitary-ovarian suppression, 112.5–300 IU/d of recombinant follicular-stimulating hormone (rFSH; Gonal-F or Puregon, Merck Serono S.A., Coinsins, Switzerland) were administered for 4–5 days.

For the GnRH antagonist protocol, ovarian stimulation started with 112.5–300 IU of rFSH from day 3 of the menstrual cycle. The dosage of rFSH was adjusted according to the ovarian response, which was assessed by ultrasound and serum hormone levels. A daily dose of 0.25 mg GnRH antagonist (Cetrotide, Serono, Switzerland) was initiated when a lead follicle reached a mean diameter of 14 mm; the dose was continued until the day of human chorionic gonadotropin (hCG) administration.

For both standard ovarian stimulation protocols, hCG was injected after confirmation of adequate follicle stimulation by ultrasound and serum hormone levels.

### Embryo Transfer Policy

Oocyte retrieval was performed 35–36 h after trigger injection. Drugs and techniques for oocyte aspiration, oocyte, and embryo culture, insemination, ICSI, assisted hatching, and embryo transfer were performed routinely (ISO 9001 Certification) (14). No more than three embryos were transferred on day 3–5 after oocyte collection. The luteal phase was supported with vaginal progesterone (Crinone 8%; Merck Serono) in all patients.

For the frozen embryo transfer cycle, no more than three embryos were transferred to each patient. Embryos were warmed using a commercially available warming solution (Kitazato Biopharma), according to the manufacturer's instruction (15). After warming, the embryos were transferred to G1.5/G2.5 medium and cultured for 2–6 h. Only embryos in the cleavage stage that exhibited >50% intact blastomeres or blastocysts that re-expanded after warming were considered as suitable for transfer. The cleavage stage embryos or blastocysts were transferred 3 or 5 days after ovulation in a natural cycle or 3 or 5 days after progesterone supplementation in hormone replacement treatment cycle. Luteal support was applied when the dominant follicle disappeared in a natural cycle or satisfactory endometrial development (thickness ≥ 8 mm, confirmed by ultrasound examination) in hormone replacement treatment cycle.

### Data Collection

Infertility is defined as the inability to conceive after 1 year of unprotected sexual intercourses (16). Duration of infertility was defined as the time from the start of regular, unprotected sexual intercourse, to enrollment. All demographic and clinical data were obtained from the database of our hospital. Follicular-stimulating hormone (FSH) levels, estradiol levels, luteinizing hormone (LH) levels, AMH levels, and AFC were measured before the first ART treatment. Serum AMH levels were measured routinely using an enzyme-linked immunosorbent assay (ELISA) kit, according to the manufacturer's instructions (KR-AMH-001; Kangrun biotech, Guangzhou, China; the antibody was from Ansh Labs, USA). The minimum detectable concentration for AMH was 0.06 ng/mL. The intra-assay coefficients of variation were <8%. AFC was defined as the number of follicles of 2–9 mm in diameter, counted at 2–5 days during menstruation using B-mode ultrasound. The maximum time between AMH assessment and the first ovarian stimulation cycle was 1 year.

Live birth was defined as a neonate showing any sign of life, irrespective of gestational age, as defined by the World Health Organization (WHO) (17).

An ovarian stimulation cycle encompassed the initiation of ovarian stimulation, aspiration, insemination, and all resulting separate fresh or frozen embryo transfers. A cycle with no oocytes retrieved after ovarian stimulation or with no embryo transfer was also considered as a stimulation cycle. Cycles canceled before oocyte retrieval were not included in the analysis.

The CLBR within one complete IVF/ICSI treatment cycle was defined as the probability of a live birth from an ovarian stimulation, including all embryo transfers (fresh and frozen) from that stimulation.

The CLBR of repeated ovarian stimulation cycles (during the study period) was defined as the probability of a live birth from all cycles during the study period. Finally, a woman who had a live birth after an ovarian stimulation cycle, regardless of fresh or frozen embryo transfer, was considered as a new patient if she underwent a new ART cycle (18, 19).

### Dealing With the Discontinuation of Assisted Reproduction Technology Treatment

Infertile women may discontinue ART treatment for physical, psychological reasons, and/or relationship problems (20). Nevertheless, CLBR assumptions (both optimal and conservative) have to take into account the rate of those who discontinued would they have pursued ART treatments. The optimal estimate is based on the observed data and assumes that the CLBR in women who discontinue ART treatment without live-birth would be equal to the rate in those who continue (21). The conservative estimate assumes that those who discontinue ART treatment would have had a live-birth rate of zero (21).

### Statistical Analysis

Continuous variables are expressed as means ± standard deviation, and were compared using one-way analysis of variance (ANOVA) and the *post-hoc* Bonferroni test. Categorical variables are expressed as frequencies (percentages), and were compared using the chi-square test or Fisher's exact test. The CLBRs within a complete IVF/ICSI treatment cycle and across all cycles, as well as the optimal and conservative estimates, were calculated. The estimated CLBR depends on the estimate of the live birth rate within each aspiration cycle (*p*) (22). It is calculated as CLBR = 1-(1-*p*_1_)(1-*p*_2_)…(1-*p*_*t*_). The optimal estimate is calculated as *p*_*t*_ = *x*_*t*_*/n*_*t*_. The conservative estimate is calculated as (*x*_1_+*x*_2_…+*x*_*t*_)/*n*_1_ (23). Univariable and multivariable logistic regression analyses were used to analyze the factors associated with live birth in the first and second stimulation cycles; odds ratios (ORs) and 95% confidence intervals (CIs) were calculated. “No live birth” was considered as the event in the logistic regression; therefore, variables with OR > 1 were risk factors (i.e., detrimental to live births) while variables with OR < 1 were protective factors (i.e., improved live birth rates). POSEIDON group 1 was taken as the reference group for CLBR prognosis. *P* < 0.05 was considered statistically significant.

## Results

### Patients

**Figure 1:**
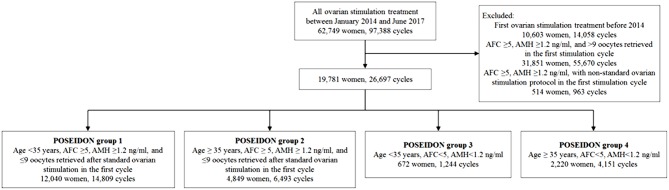
Flowchart of patient recruitment. Between January 2014 and June 2017, 62,749 women underwent ovarian stimulation. Among them, 19,781 (31.52%) fulfilled the POSEIDON criteria and were included in the study. AFC, antral follicular count; AMH, anti-Müllerian hormone.

Between January 2014 and June 2017, 62,749 women underwent ovarian stimulation at the Reproductive and Genetic Hospital of CITIC-XIANGYA. Among them, 19,781 (31.52%) fulfilled the POSEIDON criteria and were included in the study. Figure 1 presents the patient flowchart.

Table 1 shows that age, BMI, duration of infertility, baseline FSH levels, and baseline estradiol levels increased with the POSEIDON group (all *P* < 0.001), while baseline LH levels, baseline AMH levels, baseline AFC, and the frequency of ovarian hyperstimulation syndrome (OHSS) during the study period decreased with the POSEIDON group (all *P* < 0.001). The major cause of infertility in the four groups was tubal factor infertility, accounting for more than 56% of the women. Regarding the ART outcomes, the number of ovarian stimulation cycles increased with the POSEIDON grade, the number of embryo transfer cycles decreased, the number of retrieved oocytes decreased, and the number of transferred embryos decreased (all *P* < 0.001).

**Table 1:** Characteristics of the patients.

**Variable**	**POSEIDON group 1** **(*n* = 12,040)**	**POSEIDON group 2** **(*n* = 4,849)**	**POSEIDON group 3** **(*n* = 672)**	**POSEIDON group 4** **(*n* = 2,220)**	***P***
Age (years), mean ± SD	29.2 ± 3.1	38.0 ± 2.6^a^	30.6 ± 2.9^a^^b^	40.6 ± 3.3^a^^b^^c^	<0.001
BMI (kg/m^2^), *n* (%)					<0.001^*^
<18.5	1,172 (9.73)	183 (3.77)^a^	65 (9.67)^b^	56 (2.52)^a^^b^^c^	
18.5–25	9,249 (76.82)	3,749 (77.31)	523 (77.83)	1,778 (80.09)^a^	
>25	1,619 (13.45)	917 (18.91)^a^	84 (12.50)^b^	386 (17.39)^a^^c^	
Duration of infertility (years), mean ± SD	4.06 ± 2.66	6.65 ± 5.00^a^	4.61 ± 3.02^a^^b^	7.29 ± 5.77^a^^c^	<0.001
Cause of infertility, *n* (%)					<0.001^#^
Tubal	6,769 (56.22)	2,925 (60.32)^a^	382 (56.85)	1,338 (60.27)^a^	
Ovulation disorder	1 (0.01)	0 (0)	0 (0)	0 (0)	
Endometriosis	98 (0.81)	40 (0.82)	15 (2.23)^a^^b^	12 (0.54)^c^	
Male	599 (4.98)	125 (2.58)^a^	17 (2.53)^a^	67 (3.02)^a^	
Others	7 (0.06)	4 (0.08)	2 (0.30)	5 (0.23)	
Multiple causes	4,289 (35.62)	1,573 (32.44)^a^	234 (34.82)	688 (30.99)^a^	
Unknown	277 (2.30)	182 (3.75)^a^	22 (3.27)	110 (4.95)^a^	
Baseline FSH (mIU/mL), mean ± SD	6.18 ± 1.80	6.61 ± 1.98^a^	10.25 ± 6.44^a^^b^	10.5 ± 6.54^a^^b^^c^	<0.001
Baseline LH (mIU/mL), mean ± SD	4.34 ± 2.84	3.63 ± 1.79^a^	3.85 ± 3.27^a^	4.15 ± 2.92^a^^b^^c^	<0.001
Baseline E_2_ (pmol/L), mean ± SD	38.02 ± 24.05	40.23 ± 32.32^a^	51.08 ± 55.37^a^	52.79 ± 81.90^a^^b^	<0.001
Baseline AMH (ng/ml), mean ± SD	4.89 ± 3.83	2.97 ± 2.23^a^	0.56 ± 0.64^a^^b^	0.52 ± 0.64^a^^b^	<0.001
Baseline AFC, mean ± SD	20.04 ± 10.72	12.69 ± 6.91^a^	3.01 ± 1.00^a^^b^	2.83 ± 1.05^a^^b^	<0.001
OHSS during the study period, *n* (%)	18 (0.15)	2 (0.04)	0 (0)	0 (0)	0.076^#^
Number of ovarian stimulation cycles, mean ± SD	1.23 ± 0.49	1.34 ± 0.65^a^	1.85 ± 1.22^a^^b^	1.87 ± 1.31^a^^b^	<0.001
Number of embryo transfer cycles, mean ± SD	1.08 ± 0.46	1.07 ± 0.57	0.78 ± 0.68^a^^b^	0.72 ± 0.70^a^^b^^c^	<0.001
Total number of ovarian stimulation cycles	14,809	6,493	1,244	4,151	
Ovarian stimulation protocol, *n* (% per cycle)					<0.001
Long GnRH agonist protocol	12,722 (85.91)	3,984 (61.36)^a^	28 (2.25)^a^^b^	51 (1.23)^a^^b^^c^	
GnRH antagonist protocol	1,925 (13.00)	2,247 (34.61)^a^	512 (41.16)^a^^b^	1,589 (38.28)^a^^b^	
Others	162 (1.09)	262 (4.03)^a^	704 (56.59)^a^^b^	2,511 (60.49)^a^^b^^c^	
Number of oocytes retrieved, mean ± SD	7.14 ± 2.94	5.92 ± 2.85^a^	2.07 ± 2.16^a^^b^	1.65 ± 1.76^a^^b^^c^	<0.001
Method of fertilization, *n* (% per cycle)					<0.001
IVF	10,920 (73.74)	4,907 (75.57)^a^	862 (69.29)^a^^b^	2,992 (72.08)^a^^b^	
ICSI	3,889 (26.26)	1,586 (24.43)^a^	382 (30.71)^a^^b^	1,159 (27.92)^a^^b^	
Total number of embryo transfer events	15,159	6,159	560	1,695	<0.001
Number of embryos transferred, mean ± SD	1.86 ± 0.35	1.81 ± 0.42^a^	1.60 ± 0.50^a^^b^	1.52 ± 0.52^a^^b^^c^	<0.001

*Data are shown as number of patients (percentage) or mean ± SD. SD, standard deviation; BMI, body mass index; FSH, follicular-stimulating hormone; LH, luteinizing hormone; E_2_, estradiol; P, progesterone; PRL, prolactin; AMH, anti-Müllerian hormone; AFC, antral follicular count; OHSS, ovarian hyperstimulation syndrome; GnRH, gonadotropin-releasing hormone; IVF, in vitro fertilization; ICSI, intracytoplasmic sperm injection*.

a P < 0.05, vs. POSEIDON group 1;

b P < 0.05, vs. POSEIDON group 2;

c P < 0.05, vs. POSEIDON group 3;

* Chi-square test;

# *Fisher exact test*.

### Cumulative Live Birth Rates

In POSEIDON groups 1, 2, 3, and 4, the CLBRs after one complete cycle were 56.04, 30.85, 14.73, and 6.58%, respectively. After three cycles, the optimal and conservative CLBR were (from group 1 to group 4) 83.87 and 66.06%, 53.67 and 37.72%, 44.24 and 27.98%, 14.20 and 9.68%, respectively, in the four groups (Table 2). This indicates that women with adequate ovarian reserve had a better prognosis than those with low reserves. In addition, the impact of age is seen in both groups of ovarian reserve.

**Table 2:** Cumulative live birth rates according to the POSEIDON stratification.

**Cycle**	**Number of patients**	**Live birth, *n* (%)**	**CLBR across all cycles (%)**	
			**Optimal estimate^a^**	**Conservative estimate^b^**
**POSEIDON GROUP 1**				
First cycle	12,040	6,747 (56.04)	56.04	56.04
Second cycle	2,452	1,123 (45.80)	76.17	65.37
Third cycle	277	84 (30.32)	83.87	66.06
Fourth cycle	32	5 (15.63)	86.39	66.10
Fifth cycle	6	3 (50.00)	93.20	66.13
≥Sixth cycle	1	0 (0)	93.20	66.13
**POSEIDON GROUP 2**				
First cycle	4,849	1,496 (30.85)	30.85	30.85
Second cycle	1,299	301 (23.17)	46.87	37.06
Third cycle	250	32 (12.80)	53.67	37.72
Fourth cycle	64	9 (14.06)	60.17	37.90
Fifth cycle	19	0 (0)	60.17	37.90
≥Sixth cycle	9	0 (0)	60.17	37.90
**POSEIDON GROUP 3**				
First cycle	672	99 (14.73)	14.73	14.73
Second cycle	331	63 (19.03)	30.91	24.11
Third cycle	135	26 (19.26)	44.24	27.98
Fourth cycle	51	11 (21.57)	56.29	29.61
Fifth cycle	24	1 (4.17)	58.12	29.76
≥Sixth cycle	16	0 (0)	58.12	29.76
**POSEIDON GROUP 4**				
First cycle	2,220	146 (6.58)	6.58	6.58
Second cycle	1,062	55 (5.18)	11.46	9.05
Third cycle	457	14 (3.06)	14.20	9.68
Fourth cycle	202	11 (5.45)	18.83	10.18
Fifth cycle	94	3 (3.19)	21.43	10.32
≥Sixth cycle	53	4 (7.55)	25.91	10.50

*Data are shown as number of patients and percentages. CLBR, cumulative live-birth rate*.

a *The optimal estimate assumes that the CLBR in women who discontinue assisted reproductive technology treatment without live-birth would be equal to the rate in those who continue*.

b *The conservative estimate assumes that those who discontinue assisted reproductive technology treatment would have had a live-birth rate of zero*.

**Figure 2:**
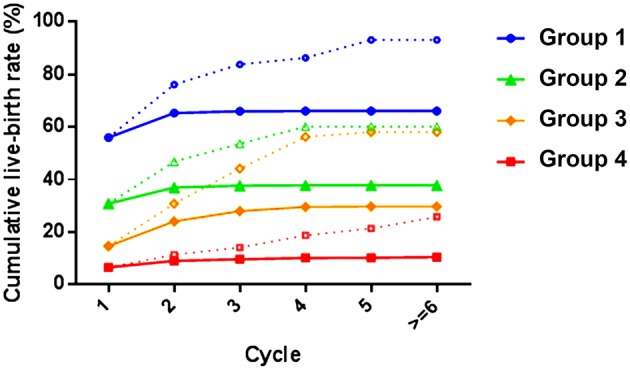
Cumulative live-birth rates according to the POSEIDON stratification. The POSEIDON groups 1 and 2 included patients with antral follicle count (AFC) ≥ 5, anti-Müllerian hormone (AMH) ≥ 1.2 ng/ml, and ≤9 oocytes retrieved after standard ovarian stimulation [long gonadotropin-releasing hormone (GnRH) agonist protocol or GnRH antagonist protocol]. Younger patients (age < 35 years) were included in POSEIDON group 1, while older patients (age ≥ 35 years) were included in POSEIDON groups 2. The POSEIDON groups 3 and 4 consisted of patients with AFC < 5 or AMH < 1.2 ng/ml. Younger patients (age < 35 years) were included in POSEIDON group 3, while older patients (age ≥ 35 years) were included in POSEIDON groups 4. The full lines are the conservative estimated live-birth rate and the dashed lines are the optimal estimated live-birth rate.

Figure 2 shows that the CLBR increased from cycle 1 to cycle 3 in all POSEIDON groups, plateauing thereafter, which is expected from multiple attempts at pregnancy. The optimal estimated CLBR of POSEIDON groups 1 and 2 differed by 30% after three ART cycles. Women in POSEIDON group 3 are young but with low ovarian reserve. If they continue ART treatment, the optimal estimated CLBR could be comparable to that of older women with adequate ovarian reserve (POSEIDON group 2). Although the CLBR of women receiving more than three cycles could not be analyzed due to the small sample size, there was such a trend observed during the first three cycles, which needs to be further studied. Taken together, significant differences in CLBR were observed among the four POSEIDON groups. Among all women, 2,841 (23.60%), 2,054 (42.36%), 242 (36.01%), and 1,012 (45.59%) discontinued ART treatment in POSEIDON groups 1, 2, 3, and 4 if they did not have a live birth after the first cycle. In addition, the number of women with two, three, and four cycles decreased, even when accounting for those with successful cycle. For women in POSEIDON groups 1, 2, and 3, the prognosis was favorable after three successive cycles of IVF/ICSI treatment.

### Factors Associated With Live Birth in the First Two Cycles

The multivariable analysis for the first and second stimulation cycles are shown in Tables 3, 4, respectively. Table 3 shows that the POSEIDON stratification (group 2: OR = 2.319, 95% CI: 2.131–2.525, *P* < 0.001; group 3: OR = 1.356, 95% CI: 1.005–1.828, *P* = 0.046; group 4: OR = 3.525, 95% CI: 2.774–4.479, *P* < 0.001; all vs. group 1), ovarian stimulation protocol (GnRH antagonist protocol: OR = 1.856, 95% CI: 1.640–2.100, *P* < 0.001; other ovarian stimulation protocols: OR = 1.651, 95% CI: 1.155–2.361, *P* = 0.006; both vs. long GnRH agonist protocol), number of embryos transferred (2: OR = 0.514, 95% CI: 0.468–0.565, *P* < 0.001), baseline LH and estradiol levels, duration of infertility, and fresh and frozen embryo transfer were independently associated with live birth in the first stimulation cycle.

**Table 3:** Logistic regression based on the occurrence of live birth in the first stimulation cycle.

**Variable**	**Non-live birth (*n* = 11,293)**	**Live birth (*n* = 8,488)**	**Univariable logistic regression**		**Multivariable logistic regression**	
			**OR (95% CI)**	***P***	**OR (95% CI)**	***P***
Grouping, *n* (%)						
POSEIDON group 1	5,293 (43.96)	6,747 (56.04)	Reference		Reference	
POSEIDON group 2	3,353 (69.15)	1,496 (30.85)	2.857 (2.662, 3.067)	<0.001	2.319 (2.131, 2.525)	<0.001
POSEIDON group 3	573 (85.27)	99 (14.73)	7.378 (5.943, 9.16)	<0.001	1.356 (1.005, 1.828)	0.046
POSEIDON group 4	2,074 (93.42)	146 (6.58)	18.104 (15.249, 21.493)	<0.001	3.525 (2.774, 4.479)	<0.001
Baseline FSH, mean ± SD	7.37 ± 3.89	6.3 ± 2.36	1.150 (1.136, 1.165)	<0.001	1.017 (1.0, 1.034)	0.053
Baseline LH, mean ± SD	4.01 ± 2.56	4.28 ± 2.79	0.962 (0.952, 0.973)	<0.001	0.979 (0.965, 0.993)	0.003
Baseline E_2_, mean ± SD	42.8 ± 45.87	37.83 ± 25.61	1.006 (1.005, 1.007)	<0.001	1.002 (1.001, 1.004)	0.001
BMI, *n* (%)						
<18.5	757 (6.70)	719 (8.47)	0.786 (0.706, 0.875)	<0.001	1.014 (0.891, 1.154)	0.831
18.5–25	8,759 (77.56)	6,540 (77.05)	Reference		Reference	
>25	1,777 (15.74)	1,229 (14.48)	1.080 (0.997, 1.169)	0.059	1.033 (0.938, 1.138)	0.505
Duration of infertility, mean ± SD	5.57 ± 4.47	4.42 ± 3.25	1.079 (1.071, 1.088)	<0.001	1.023 (1.013, 1.033)	<0.001
Cause of infertility, *n* (%)						
Tubal cause	10,309 (91.29)	7,762 (91.45)	0.98 (0.886, 1.083)	0.692		
Ovulation disorder	58 (0.51)	83 (0.98)	0.523 (0.373, 0.732)	<0.001	1.049 (0.722, 1.524)	0.803
Endometriosis	1,078 (9.55)	777 (9.15)	1.047 (0.95, 1.154)	0.351		
Male cause	3,368 (29.82)	2,816 (33.18)	0.856 (0.806, 0.909)	<0.001	0.991 (0.920, 1.068)	0.817
Others	193 (1.71)	133 (1.57)	1.092 (0.874, 1.364)	0.44		
Multiple causes	10,911 (96.62)	8,279 (97.54)	0.721 (0.608, 0.856)	<0.001	0.932 (0.758, 1.146)	0.502
Method of fertilization, *n* (%)						
IVF	8,853 (78.39)	6,712 (79.08)	Reference			
ICSI	2,440 (21.61)	1,776 (20.92)	1.042 (0.972, 1.116)	0.247		
Ovarian stimulation protocol, *n* (%)						
Long GnRH agonist protocol	7,192 (63.69)	7,773 (91.58)	Reference		Reference	
GnRH antagonist protocol	2,630 (23.29)	653 (7.69)	4.353 (3.972, 4.77)	<0.001	1.856 (1.640, 2.100)	<0.001
Others	1,471 (13.03)	62 (0.73)	25.642 (19.848, 33.128)	<0.001	1.651 (1.155, 2.361)	0.006
Fresh embryo/frozen embryo, *n* (%)						
Fresh embryo transfer	7,510 (66.50)	6,927 (81.61)	Reference		Reference	
Fresh and frozen embryo transfer	3,783 (33.50)	1,561 (18.39)	2.235 (2.089, 2.39)	<0.001	2.772 (2.567, 2.993)	<0.001
Number of embryos transferred, *n* (%)						
0	3,998 (35.4)	0 (0)	>999.999 (0, >999.999)	0.903	>999.999 (0, >999.999)	0.973
1	1,938 (17.16)	945 (11.13)	Reference		Reference	
2	5,296 (46.9)	7,503 (88.4)	0.344 (0.316, 0.375)	<0.001	0.514 (0.468, 0.565)	<0.001
3	61 (0.54)	40 (0.47)	0.744 (0.495, 1.116)	0.153	0.747 (0.483, 1.155)	0.190

*Data are shown as number of patients (percentage) or mean ± SD. OR, odd ratio; CI, confidence interval; BMI, body mass index; FSH, follicular-stimulating hormone; SD, standard deviation; LH, luteinizing hormone; E_2_, estradiol; P: progesterone; PRL, prolactin; AMH, anti-Müllerian hormone; AFC, antral follicular count; OHSS, ovarian hyperstimulation syndrome; GnRH, gonadotropin-releasing hormone; IVF, in vitro fertilization; ICSI, intracytoplasmic sperm injection*.

**Table 4:** Logistic regression based on the occurrence of live birth in the second stimulation cycle.

**Variable**	**Non-live birth (*n* = 2,085)**	**live birth (*n* = 1,542)**	**Univariable logistic regression**		**Multivariable logistic regression**	
			**OR (95% CI)**	***P***	**OR (95% CI)**	***P***
Grouping, *n* (%)						
POSEIDON group 1	1,328 (36.88)	1,123 (72.83)	Reference		Reference	
POSEIDON group 2	998 (27.71)	301 (19.52)	2.804 (2.41, 3.262)	<0.001	2.098 (1.762, 2.497)	<0.001
POSEIDON group 3	268 (7.44)	63 (4.09)	3.597 (2.703, 4.787)	<0.001	0.952 (0.649, 1.397)	0.803
POSEIDON group 4	1,007 (27.96)	55 (3.57)	15.478 (11.666, 20.536)	<0.001	3.887 (2.726, 5.541)	<0.001
Baseline FSH, mean ± SD	8.02 ± 4.59	6.57 ± 2.5	1.143 (1.117, 1.17)	<0.001	1.011 (0.984, 1.038)	0.440
Baseline LH, mean ± SD	4.03 ± 2.56	4.14 ± 2.49	0.983 (0.961, 1.006)	0.145		
Baseline E_2_, mean ± SD	44.89 ± 41.83	38.23 ± 25.85	1.007 (1.005, 1.01)	<0.001	1.004 (1.001, 1.006)	0.012
BMI, *n* (%)						
<18.5	207 (5.75)	105 (6.81)	0.846 (0.663, 1.079)	0.178		
18.5–25	2,961 (82.23)	1,270 (82.36)	Reference			
>25	433 (12.02)	167 (10.83)	1.112 (0.919, 1.345)	0.274		
Duration of infertility, mean ± SD	5.86 ± 4.59	4.79 ± 3.53	1.078 (1.062, 1.094)	<0.001	1.022 (1.003, 1.041)	0.025
Cause of infertility, *n* (%)						
Tubal cause	3,204 (88.98)	1,344 (87.16)	1.189 (0.991, 1.426)	0.062		
Ovulation disorder	6 (0.17)	4 (0.26)	0.642 (0.181, 2.277)	0.492		
Endometriosis	282 (7.83)	149 (9.66)	0.794 (0.645, 0.978)	0.03	0.828 (0.645, 1.063)	0.139
Male cause	819 (22.74)	401 (26.01)	0.838 (0.73, 0.962)	0.012	0.956 (0.813, 1.123)	0.582
Others	71 (1.97)	21 (1.36)	1.456 (0.892, 2.379)	0.133		
Multiple causes	3,412 (94.75)	1,462 (94.81)	0.988 (0.755, 1.292)	0.929		
Method of fertilization, *n* (%)						
IVF	2,205 (61.23)	909 (58.95)	Reference			
ICSI	1,396 (38.77)	633 (41.05)	0.909 (0.805, 1.027)	0.125		
Ovarian stimulation protocol, *n* (%)						
Long GnRH agonist protocol	866 (24.05)	833 (54.02)	Reference		Reference	
GnRH antagonist protocol	1,672 (46.43)	611 (39.62)	2.632 (2.305, 3.006)	<0.001	1.761 (1.416, 2.189)	<0.001
Others	1,063 (29.52)	98 (6.36)	10.434 (8.309, 13.102)	<0.001	1.767 (1.252, 2.494)	0.001
Change of ovarian stimulation protocol, *n* (%)						
No	1,866 (51.82)	928 (60.18)	Reference		Reference	
Yes	1,735 (48.18)	614 (39.82)	1.405 (1.245, 1.586)	<0.001	0.876 (0.715, 1.074)	0.204
Fresh embryo/frozen embryo, *n* (%)						
Fresh embryo transfer	2,126 (59.04)	895 (58.04)	Reference			
Fresh and frozen embryo transfer	1,475 (40.96)	647 (41.96)	0.96 (0.85, 1.083)	0.505		
Number of embryos transferred, *n* (%)						
0	1,516 (42.1)	0 (0)	>999.999 (0, >999.999)	0.917	>999.999 (0, >999.999)	0.984
1	643 (17.86)	225 (14.59)	Reference		Reference	
2	1,439 (39.96)	1,313 (85.15)	0.384 (0.324, 0.454)	<0.001	0.518 (0.432, 0.621)	<0.001
3	3 (0.08)	4 (0.26)	0.262 (0.058, 1.182)	0.093	0.255 (0.052, 1.257)	0.093

*Data are shown as number of patients (percentage) or mean ± SD. OR, odd ratio; CI, confidence interval; BMI, body mass index; FSH, follicular-stimulating hormone; SD, standard deviation; LH, luteinizing hormone; E_2_, estradiol; P, progesterone; PRL, prolactin; AMH, anti-Müllerian hormone; AFC, antral follicular count; OHSS, ovarian hyperstimulation syndrome; GnRH, gonadotropin-releasing hormone; IVF, in vitro fertilization; ICSI, intracytoplasmic sperm injection*.

Table 4 shows that the POSEIDON stratification (group 2: OR = 2.098, 95% CI: 1.762–2.497, *P* < 0.001; group 4: OR = 3.887, 95% CI: 2.726–5.541, *P* < 0.001; both vs. group 1), ovarian stimulation protocol (GnRH antagonist protocol: OR = 1.761, 95% CI: 1.416–2.189, *P* < 0.001; other ovarian stimulation protocols: OR = 1.767, 95% CI: 1.252–2.494, *P* = 0.001; both vs. long GnRH agonist protocol), number of embryos transferred (2: OR = 0.518, 95% CI: 0.432–0.621, *P* < 0.001), baseline estradiol levels, and duration of infertility were independently associated with live birth in the second stimulation cycle. Change of ovarian stimulation protocol was not independently associated with improvement in live birth rate.

## Discussion

The POSEIDON criteria are used to stratify women with low prognosis after ART treatment. To the best of our knowledge, this is the first large population study that analyzed the CLBRs of women according to POSEIDON groups. The conservative CLBR was the highest for POSEIDON group 1, followed by groups 2, 3, and 4. Unsurprisingly, the results show that it is indeed more difficult for older women with POR to achieve a live birth after IVF/ICSI compared with younger women. Age is the key factor influencing CLBR after ART (24–26).

In women with POR according to the Bologna criteria, the CLBR within one cycle was previously reported to be poor (27), and the overall prognosis was improved after three cycles (conservative CLBR: from 10.5 to 14.3; optimal CLBR: from 10.5 to 23.7) (26). When using the Bologna criteria, it is difficult to distinguish POR women with differences in reproductive prognosis. In contrast, the POSEIDON criteria were specifically designed to solve the heterogeneity observed in the Bologna criteria (7, 8, 12, 13).

POSEIDON groups 1 and 2 are characterized by hypo-response to ovarian stimulation, which can be caused by among others environmental contaminants, polymorphisms, and drugs. The mechanisms are still far from being understood, but studies suggest some genetic causes (28–30). A study showed that intrafollicular concentrations of benzene were associated with the outcomes of ART treatment, suggesting that environmental or occupational exposure to pollutants affects the reproductive outcomes (31). Women in POSEIDON groups 1 and 2 have an adequate ovarian reserve, but unexpectedly the ovarian response to stimulation is either poor or suboptimal, defined as <4 oocytes and ≤9 oocytes, respectively. Dose adjustments of FSH in the subsequent cycles and possible supplementation with recombinant LH might help these patients produce more follicles and oocytes (32, 33). Regarding POSEIDON groups 3 and 4, the low AFC and the expected decrease in the number of euploid embryos for transfer are the main causes of poor outcomes. The retrieval of a large number of oocytes (which will increase the likelihood of having at least one euploid embryo) is difficult in POSEIDON 3 and 4 women (34). In POSEIDON groups 3 and 4, the reasons for poor response include poor ovarian reserve, asynchronous development, and genetic polymorphisms in FSH receptor, LH receptor, and the possible presence of variant LH-β. The clinical management include down-regulation with a long GnRH agonist protocol, stimulation with recombinant FSH with or without recombinant LH, possible pre-treatment with androgens, fresh embryo transfer, or oocyte/embryo accumulation and frozen embryo transfer (34). Specifically for POSEIDON group 4, recombinant LH can be added to increase circulating androgens (which are decreased in older patients and play an important role for optimal folliculogenesis stimulation) (34).

Based on the present study, for women with an adequate ovarian reserve, but initial poor response, and who underwent three ART cycles, the optimal estimated CLBR can be up to 83.9 and 53.7%, respectively in POSEIDON groups 1 and 2. This should encourage POSEIDON groups 1 and 2 women to pursue cycles when unsuccessful. Unfortunately, the number of women who received more than three cycles was too small to perform any reliable analysis. Nevertheless, Gu et al. (35) and Smith et al. (23) previously showed that CLBR increases with repeated ART in younger women.

In the present study, CLBR was not only associated with the POSEIDON stratification, but also with clinical factors such as duration of infertility and ovarian stimulation protocols. This is generally consistent with the literature (36, 37). Interestingly, compared with the first stimulation cycle, changing the ovarian stimulation protocol in the second cycle did not seem to be associated with an improvement in live birth. Due to the retrospective nature of this study, results need to be confirmed by prospective studies.

It is quite clear that natural cycle IVF results in a very low CLBR (1.2%) in women with POR according to the Bologna criteria (at least two of the following criteria: ≥40 years of age; history of <3 oocytes with a conventional ovarian stimulation method; and AFC < 5.7) (26). In contrast, ovarian stimulation with exogenous gonadotropins results in a significantly higher live birth rate after one stimulation with fresh embryo transfer when using the long GnRH agonist protocol (11.7%) (38) or the GnRH antagonist protocol (15–20%) (39–41).

The long GnRH agonist protocol was used for most women in POSEIDON groups 1 and 2, while the GnRH antagonist protocol was used for women in POSEIDON groups 3 and 4. Although the long GnRH agonist protocol is used to a lower extent in other countries (42, 43), it is still used as the preferred protocol in China (44–46). In the present study, although changing the ovarian stimulation protocol during the second cycle did not seem to improve the live birth rate, selecting different ovarian stimulation protocols were associated with live birth. Further studies should be conducted to explore the impact of different ovarian stimulation protocols in the POSEIDON population. Considering the poor baseline condition of these women, individualized treatment in all steps of ART, including the choice of gonadotropin type and dose, ovulation trigger, and the possible use of adjuvant therapies should be considered (34). Importantly, the most optimal protocol for POSEIDON groups 3 and 4 still remains a clinical challenge.

A limitation of this study is its retrospective nature. Nevertheless, this is the largest study so far (26,697 cycles) assessing the prevalence, management, and effect of the POSEIDON classification in a Chinese IVF population. In addition, Humaidan et al. (7) showed that the POSEIDON criteria can be applied retrospectively to structured databases to determine the POSEIDON groups. Of course, the treatment that the patient ultimately received was not tailored according to the POSEIDON groups because of the retrospective determination of grouping, and the subsequent reproductive outcomes in relation to POSEIDON must be taken with caution. These results will have to be confirmed prospectively. Furthermore, there might be ethnic differences, which could impair the generalizability of the present study to other populations. Nevertheless, our data are in line with previous smaller reports from other regions of the world (12, 13). Future prospective studies in different populations may be necessary to validate the results. Secondly, we stratified patients according to the baseline data only, and a re-stratification of patients during the subsequent stimulation cycles was not performed. Finally, no data about the dose and type of gonadotropins used for ART treatments were collected. Further studies are essential to explore whether adjustment should be performed and whether this would further improve the outcome of the POSEIDON patient.

In conclusion, our study showed that more than 30% of Chinese women undergoing IVF/ICSI treatment can be classified into one of the four POSEIDON groups, and that significant differences in reproductive prognosis are observed between the four POSEIDON groups. Patients with low prognosis may increase their chances of live birth by repeated ART treatments. For women in POSEIDON groups 1, 2, and 3, the prognosis was favorable after three successive cycles of IVF/ICSI treatment. Future randomized controlled trials should investigate whether the CLBR of the low prognosis patient can be improved by individualized ART treatment according to the suggestions made by the POSEIDON stratification group.

## Data Availability Statement

All datasets generated for this study are included in the manuscript/supplementary files.

## Author Contributions

YL conceived and coordinated the study, designed, performed, and analyzed the experiments, and wrote the paper. XL, XY, and SC carried out the data collection and data analysis. GLu and GLi designed and guided the study. PH supervised and revised the paper. FG designed and guided the study. All authors reviewed the results and approved the final version of the manuscript.

### Conflict of Interest

The authors declare that the research was conducted in the absence of any commercial or financial relationships that could be construed as a potential conflict of interest.
